# TYROSINE KINASE INHIBITOR INDUCED PERICARDIAL EFFUSION IN ISOLATION

**DOI:** 10.51894/001c.123107

**Published:** 2024-08-30

**Authors:** Stephen Caucci

**Affiliations:** 1 McLaren Greater Lansing

## 88

### INTRODUCTION

Modern treatment for malignancy involves tailor-made treatments for specific receptors and mutations in a multitude of different malignancies. One of the most prominent examples is the advent of tyrosine kinase inhibitors (TKIs) in the treatment of BCR-ABL positive malignancies. As these become more standard care, side effects need to be discussed more prominently.

### CASE DESCRIPTION

A 71-year-old male with chronic myeloid leukemia (CML) on dasatinib 100 mg daily for the past 2.5 years was transferred from an outside facility for right flank pain and a moderate to large pericardial effusion was found on CT. He was treated medically and a repeat echocardiogram confirmed improvement in the effusion size. A follow-up limited echocardiogram revealed a large effusion with a maximum depth of 2.6 cm. He underwent pericardiocentesis and 450 cc of serous fluid was removed. He was monitored in the hospital, started on colchicine, and a follow-up echocardiogram outpatient demonstrated no evidence of residual pericardial effusion. A month later, bosutinib 400 mg daily was initiated and a pericardial effusion returned as well as pleural effusion; both of which required drainage including the creation of a pericardial window. Given the stability of lab work, length of treatment, and recent complications of TKIs, the decision was made to withhold TKI treatment.

### DISCUSSION/CONCLUSION

Here, we present a patient with an isolated pericardial effusion attributed to TKI use, which is the only second ever reported to our knowledge. This case helps to stress the importance for providers to be aware of monitoring for isolated pericardial effusions while patients are on TKIs. Future research should be dedicated to creating a protocol in which patients may benefit from screening before symptoms start.

